# Supplementary material for: The Role of Human Chorionic Gonadotropin Beta (hCGβ) in HPV-Positive and HPV-Negative Oropharyngeal Squamous Cell Carcinoma
Source: Cancers (Basel). 2022 Jun 8;14(12):2830. doi: 10.3390/cancers14122830 (PMC9221036; doi:10.3390/cancers14122830)
Supplement: Supplementary file 1 [file cancers-14-02830-s001.zip › cancers-1738595-supplementary.pdf]

Table S1. Clinicopathological data according to hCGβ-expression in the tumor tissue. Abbreviations: hCGβ: Human chorionic gonadotropin; HPV: Human papillomavirus. hCGβ immunoexpression was scored in the nuclei of the tumor tissue.

Variable	hCGβ Positivity in Tumor Tissue	%	hCGβ Negativity in Tumor Tissue	%	*p*-Value	Missing/ % (n=18)
Number of patients	12	66.7	6	33.3		
Mean age at diagnosis	66.1		63.5		0.854	
Gender						
Male	8	66.7	4	33.3		
Female	4	66.7	2	33.3	1.000	
Smoking						
Non-smoker	2	50.0	2	50.0		
Ex-smoker	3	60.0	2	40.0		
Current smoker	7	77.8	2	22.2	0.548	
Heavy alcohol use						3 / 16.7
Non-drinker	5	83.3	1	16.7		
Ex-drinker	1	50.0	1	50.0		
Current drinker	5	71.4	2	28.6	0.769	
T class						
T1–T2 56	8	57.1	6	42.9		
T3–T4	4	100.0	0	0.0	0.245	
N class						
N0–N1	8	61.5	5	38.5		
N2–N3	4	80..0	1	20.0	0.615	
Stage						
I–II	6	54.5	5	45.5		
III–IV	6	85.7	1	14.3	0.316	
Grade						
I	7	87.5	1	12.5		
II	5	50.0	5	50.0		
III	12	66.7	6	33.3	0.094	
Localization						
Tonsil	6	60.0	4	40.0		
Base of tongue	2	66.7	1	33.3		
Soft palate	3	75.0	1	25.0		
Posterior wall of oropharynx	1	100.0	0	0.0	1.000	
HPV						
HPV+	4	50.0	4	50.0		
HPV-	8	80.0	2	20.0	0.321	
S- hCGβ						
S-hCGβ+	4	33.3	8	66.7		
S-hCGβ-	1	16.7	5	83.3	0.615	
